# Reducing False Negatives in AI-Based Breast Histopathology: A Clinically Oriented Evaluation of Deep Learning Models Under Domain Shift

**DOI:** 10.3390/diagnostics16152371

**Published:** 2026-07-28

**Authors:** Liana Stanescu, Cosmin Stoica Spahiu

**Affiliations:** Department of Computer Science and Information Technology, Faculty of Automation, Computers and Electronics, University of Craiova, 200585 Craiova, Romania; cosmin.spahiu@edu.ucv.ro

**Keywords:** breast cancer, histopathology, domain shift, transfer learning, false-negative analysis, deep learning, ConvNeXt, Swin Transformer, MaxViT, deployment-oriented evaluation

## Abstract

**Background/Objectives:** Deep learning approaches have demonstrated strong performance in breast histopathology image classification; however, reliable generalization across heterogeneous acquisition environments remains challenging due to domain shift. In clinical practice, missed malignant cases are particularly critical because they may directly affect diagnostic decisions and patient outcomes. This study systematically investigates the behavior of modern deep learning architectures and adaptation strategies under realistic cross-domain conditions, with particular emphasis on malignant case detection and false-negative reduction. **Methods:** Three modern architectures—ConvNeXt-Tiny, Swin-Tiny, and MaxViT-Tiny—were initially trained on a large-scale breast histopathology dataset and subsequently evaluated on the BreaKHis dataset using strict patient-level separation to avoid information leakage. Three transfer settings were investigated: direct zero-shot transfer, head-only adaptation, and full fine-tuning. Performance was evaluated independently across four magnification levels (40×, 100×, 200×, and 400×) using accuracy, precision, sensitivity, F1-score, ROC–AUC, PR–AUC, and false-negative rates. **Results:** Direct zero-shot transfer produced substantial performance degradation across all architectures, with mean false-negative rates ranging from 75.85% to 90.11%, highlighting the limited transferability of source-domain representations under heterogeneous acquisition conditions. Both adaptation strategies substantially improved performance and reduced missed malignant cases to below 10%. Swin-Tiny under head-only adaptation achieved the most favorable malignant detection profile, reaching a mean sensitivity of 97.36% while reducing the average false-negative rate to 2.64%. In contrast, MaxViT-Tiny achieved the highest mean ROC–AUC value (0.849) after full fine-tuning, although this did not correspond to the lowest false-negative burden. **Conclusions:** The findings demonstrate that maximizing global discrimination performance does not necessarily correspond to optimal malignant detection under cross-domain conditions. Sensitivity and missed-case analysis provide complementary information beyond conventional discrimination metrics and may support more informed model assessment. Furthermore, the proposed methodology provides a reproducible framework for investigating adaptation performance in AI-assisted breast histopathology systems.

## 1. Introduction

Breast cancer remains one of the most frequently diagnosed malignancies worldwide and continues to represent a major cause of cancer-related mortality among women [1]. Early detection and accurate diagnosis are critical for improving patient outcomes and reducing mortality rates. Histopathological examination of tissue biopsies remains the reference standard for breast cancer diagnosis because it enables detailed assessment of tissue morphology and direct microscopic evaluation of cellular architecture, allowing reliable differentiation between benign and malignant lesions. In contrast, imaging modalities such as ultrasound primarily provide macroscopic structural information and are commonly used as complementary diagnostic tools rather than definitive pathological assessment methods. However, manual examination of histopathological slides is labor-intensive, time-consuming, and may be affected by inter-observer variability, particularly in cases presenting subtle morphological differences. Thus, considerable effort has been directed toward developing computer-aided diagnosis (CAD) systems capable of supporting pathologists and improving diagnostic consistency.

Recent progress in deep learning has significantly transformed medical image analysis and breast cancer imaging applications [2,3]. Convolutional neural networks (CNNs) have demonstrated strong capability in automatically learning hierarchical representations directly from image data and have achieved promising performance across multiple breast imaging and histopathological analysis tasks [2,3]. More recently, transformer-based architectures have attracted increasing attention due to their ability to capture long-range contextual relationships within tissue structures. Modern architectures such as ConvNeXt, Swin Transformer, and MaxViT have demonstrated competitive performance across multiple computer vision and medical imaging applications [4,5,6].

Despite these advances, one of the major challenges limiting the broader adoption of artificial intelligence systems in pathology is their reduced ability to generalize across heterogeneous acquisition environments. Models frequently demonstrate excellent performance when evaluated on data originating from the same source used during training; however, performance may substantially deteriorate when the same systems are applied to images acquired at different institutions [7]. This phenomenon, commonly referred to as domain shift, may arise from differences in staining procedures, slide preparation protocols, scanner characteristics, image acquisition settings, and patient populations. Consequently, strong source-domain performance does not necessarily indicate reliable behavior when models are applied to previously unseen data distributions [7].

Another important methodological issue concerns the evaluation strategy employed in computational pathology studies. Several breast histopathology investigations based on publicly available datasets, including BreaKHis [8], have traditionally relied on image-level evaluation protocols, potentially leading to optimistic estimates of model performance. Such practices may introduce information leakage because images from the same patient may appear simultaneously in training and test subsets. In realistic applications, however, diagnostic systems encounter entirely unseen patients rather than previously observed image patches. Therefore, patient-level separation provides a more reliable framework for assessing model generalization and reducing the risk of overly optimistic performance estimates.

Beyond aggregate classification performance, additional concerns emerge regarding the interpretation of model behavior from a diagnostic perspective. Conventional metrics such as accuracy and ROC–AUC provide useful information regarding global discrimination capability. However, diagnostic error profiles, particularly missed malignant cases, have increasingly attracted attention in breast pathology applications [9,10]. As a result, minimizing false-negative errors may represent an important complementary objective alongside maximizing discrimination performance.

Although transfer learning and AI-based pathology systems have demonstrated considerable potential, important challenges regarding robustness and practical applicability remain [9,10]. Existing studies frequently investigate a single adaptation strategy and primarily emphasize conventional performance metrics. Relatively few studies systematically analyze how different adaptation depths influence sensitivity-oriented performance and false-negative rates under domain-shift conditions. Furthermore, analyses focusing on diagnostic error profiles remain comparatively limited.

To address these limitations, this study introduces a cross-domain evaluation framework for breast histopathology classification. The proposed framework explicitly prioritizes the identification of malignant cases through sensitivity-oriented analysis while employing strict patient-level separation to avoid information leakage. Within this framework, three modern deep learning architectures—ConvNeXt-Tiny [4], Swin-Tiny [5], and MaxViT-Tiny [6]—were systematically evaluated under heterogeneous domain conditions. Three transfer settings were investigated, including direct zero-shot transfer, head-only adaptation, and full fine-tuning. Furthermore, validation-based threshold optimization was incorporated to investigate its impact on sensitivity, false-negative rates, and overall model performance.

The main contributions of this work are summarized as follows:

- We introduce a cross-domain evaluation framework that combines patient-level assessment, adaptation-depth analysis, and threshold optimization for breast histopathology classification.
- We employ strict patient-level separation to eliminate information leakage and provide more realistic estimates of model generalization performance.
- We provide a systematic patient-level comparison of ConvNeXt-Tiny, Swin-Tiny, and MaxViT-Tiny under identical adaptation protocols.
- We analyze the effect of adaptation depth through zero-shot transfer, head-only adaptation, and full fine-tuning while examining its impact on sensitivity and false-negative rates.
- We evaluate optimized decision thresholds and quantify their influence on the detection of malignant samples and false-negative reduction under domain-shift conditions.

## 2. Related Work

Deep learning has substantially transformed breast histopathology analysis by enabling automated extraction of complex tissue representations and reducing dependence on handcrafted feature engineering. Recent reviews highlighted that convolutional neural networks and transformer-based approaches have become dominant paradigms for breast cancer image analysis, supporting applications ranging from lesion classification and diagnosis to prognosis prediction and decision-support applications [11,12].

Several publicly available datasets have played an important role in the development and evaluation of breast histopathology classification systems. Among these, BreaKHis remains one of the most frequently used benchmarks because it provides microscopy images acquired at multiple magnification levels (40×–400×), enabling assessment of scale-dependent model behavior [8]. The availability of large image repositories and whole-slide image collections has further facilitated the adoption of transfer-learning approaches and increasingly sophisticated feature representations in computational pathology applications [11,12].

Recent investigations reported promising performance using deep learning approaches specifically designed for breast cancer diagnosis and histopathological image analysis [13]. Furthermore, artificial intelligence systems have expanded beyond conventional image classification tasks and increasingly support prognosis prediction, grading, biomarker assessment, and clinical decision-support applications [14]. Despite these advances, many studies primarily emphasized improvements in conventional discrimination metrics while providing comparatively limited analysis of deployment robustness and diagnostic error profiles.

Reliable deployment across institutions remains challenging because histopathological images may vary substantially due to differences in staining procedures, scanner characteristics, tissue preparation protocols, and patient populations [7]. Several approaches, including stain normalization, domain adaptation, and multi-center learning strategies, have been proposed to improve robustness and generalization [15,16]. However, many of these methods introduce additional preprocessing requirements or increased computational complexity.

Earlier investigations primarily relied on conventional convolutional neural networks trained directly on breast histopathological images. Such studies demonstrated that deep learning models could successfully capture diagnostically relevant tissue characteristics and provide competitive classification performance [17]. Magnification-independent classification approaches further highlighted the importance of multi-scale information and improved robustness across different image magnifications [18]. Nevertheless, many early investigations relied on image-level data separation and focused predominantly on maximizing overall predictive performance rather than evaluating generalization under realistic deployment conditions.

More recently, computational pathology research has increasingly shifted toward weakly supervised and foundation-model-based approaches capable of learning richer and more generalizable feature representations from large-scale image collections [19,20]. These methods demonstrated encouraging performance across several computational pathology applications. However, their reliance on large-scale training resources and substantial computational requirements may limit practical feasibility for deployment-oriented settings where computational efficiency and adaptation flexibility remain important considerations.

Domain generalization remains a major challenge in digital pathology because models trained under a specific acquisition setting often experience considerable performance degradation when applied to external datasets [21]. Recent studies have shown that augmentation-based robustness strategies can reduce the impact of acquisition variability and improve generalization across heterogeneous imaging environments [22]. Nevertheless, achieving reliable performance after transfer to previously unseen datasets remains difficult, particularly when deployment involves different acquisition protocols, institutions, and patient populations. Recent work has also investigated cross-magnification robustness in breast histopathology using modern deep learning architectures and transfer-learning strategies, demonstrating that architectural design and adaptation depth can significantly affect sensitivity and false-negative rates under heterogeneous imaging settings [23].

Although conventional metrics such as accuracy and ROC–AUC remain important, they do not fully characterize diagnostic error profiles. Missed malignant cases are particularly relevant because they may directly affect patient management and treatment decisions [1,9,10]. Nevertheless, comparatively few studies have systematically investigated sensitivity-oriented performance and false-negative behavior in cross-domain breast histopathology evaluation.

To provide a concise overview of representative developments in breast histopathology analysis, Table 1 summarizes selected studies covering benchmark datasets, classification methods, robustness strategies, and deployment-related challenges.

To address this gap, the present study compares zero-shot transfer, head-only adaptation, and full fine-tuning using ConvNeXt-Tiny, Swin-Tiny, and MaxViT-Tiny within a strict patient-level evaluation framework. The analysis extends beyond conventional performance metrics by examining sensitivity, false-negative rates, and the effect of threshold optimization on model behavior.

## 3. Materials and Methods

### 3.1. Generalized Workflow for Histopathological Image Classification

To provide methodological context, Algorithm 1 summarizes a generalized deep learning workflow for histopathological image classification. The workflow is intentionally independent of the specific datasets and architectures investigated in this study and highlights the common stages encountered in computational pathology pipelines.
**Algorithm 1.** Generalized workflow for histopathological image classification1. Load source-domain dataset 2. Preprocess images 3. Train source-domain model 4. Evaluate source-domain performance 5. Apply cross-domain transfer 6. Perform adaptation 7. Optimize decision threshold 8. Evaluate on independent target data 9. Compute performance metrics 10. Perform statistical analysis 11. Analyze uncertainty characteristics

### 3.2. Proposed Training and Evaluation Procedure

Algorithm 2 summarizes the experimental pipeline used in this study, including source-domain training, target-domain adaptation, threshold optimization, and magnification-specific evaluation. To reduce information leakage and obtain a more realistic estimate of generalization, all experiments were conducted using strict patient-level separation. The pipeline begins with source-domain training on the Kaggle IDC dataset, followed by adaptation and evaluation on the BreaKHis dataset using multiple transfer strategies. Performance was subsequently assessed across all magnification levels using conventional classification metrics and clinically oriented error analysis.
**Algorithm 2.** Proposed training and evaluation procedure**Input**:        Source dataset:               Dsource = Kaggle IDC dataset        Target dataset:               Dtarget = BreaKHis        Architectures:               A = {ConvNeXt-Tiny,                        Swin-Tiny,                        MaxViT-Tiny}        Magnifications:               M = {40×, 100×, 200×, 400×} Stage 1: Source-domain training        for each architecture a ∈ A do               Initialize model using                        ImageNet pretrained weights               Replace classification head               Train model on:                        Dsource[train]               Validate on:                        Dsource[val]               Select best checkpoint a*        end for Stage 2: Cross-domain adaptation        for each architecture a* do               Apply:                        Zero-shot:                               Direct evaluation without retraining                        Head-only adaptation:                               Freeze backbone layers                               Train classification head only                        Full fine-tuning:                               Train all network parameters               Select best model according to:                        Validation ROC–AUC        end for Stage 3: Magnification-specific evaluation        for each magnification m ∈ M do               Evaluate model on:                        Dtarget[test,m]               For adapted models:                        Evaluate validation probabilities                        across threshold values t ∈ [0, 1]                        Select:                               t* = threshold maximizing sensitivity subject to specificity ≥ 70%                        Apply selected threshold during testing               For zero-shot experiments:                        Apply standard threshold:                               t = 0.5               Compute:                        Accuracy                        Precision                        Recall                        F1-score                        ROC–AUC                        PR–AUC                        False-negative rate        end for
**Output**:        Comparative performance results        Clinically oriented error analysis        Architecture comparison

**Figure 1:**
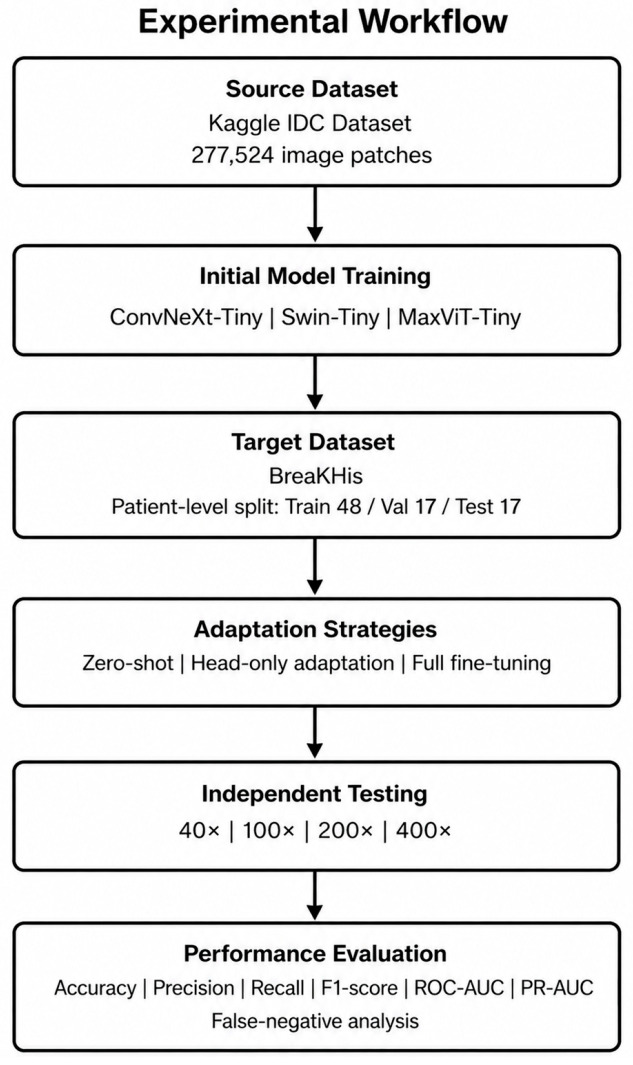
Overview of the experimental pipeline, including source-domain training, cross-domain adaptation, patient-level evaluation, and performance analysis.

Figure 1 provides a visual overview of the experimental pipeline.

### 3.3. Data Sets

This study employed two independent breast histopathology datasets to investigate model adaptation and generalization behavior under realistic cross-domain conditions. A publicly available breast histopathology dataset distributed through Kaggle [24] was used as the source domain for initial model training, while BreaKHis [8] served as the target domain for adaptation and evaluation.

The source-domain dataset contains 277,524 hematoxylin and eosin (H&E)-stained image patches of size 50 × 50 pixels, extracted from 162 whole-slide images scanned at 40× magnification, including 198,738 invasive ductal carcinoma (IDC)-negative and 78,786 IDC-positive samples [24]. Patient identifiers provided with the dataset were used to construct patient-level training, validation, and testing partitions, resulting in 279 distinct patient IDs across the entire dataset. The images originate from invasive ductal carcinoma whole-slide histopathology specimens and have been widely used for breast cancer image analysis studies [25]. To ensure compatibility with pretrained architectures, all images were resized to 224 × 224 pixels and normalized using ImageNet statistics before model training.

Source-domain partitioning was performed at the patient level. The dataset was divided into training, validation, and testing subsets containing 196, 42, and 41 patients, respectively. Patients were assigned exclusively to a single subset, ensuring that no patient appeared in more than one partition and preventing information leakage between source-domain subsets.

The target domain employed the BreaKHis dataset [8], which contains 7909 microscopic breast tumor images collected from 82 patients, acquired under four magnification levels (40×, 100×, 200×, and 400×). The dataset includes both benign and malignant tissue categories and represents one of the most frequently used benchmarks in breast histopathology research. The dataset comprises 2480 benign and 5429 malignant images.

Although the source and target tasks are not identical, both involve breast cancer malignancy assessment. The Kaggle dataset focuses on IDC-positive versus IDC-negative image patches, whereas BreaKHis addresses benign-versus-malignant tumor classification across multiple magnification levels. The transfer setting involves both domain shift and task mismatch, creating a challenging evaluation scenario. This mismatch may partially contribute to the performance degradation observed during zero-shot transfer.

For the BreaKHis dataset, patients were assigned exclusively to the training, validation, or test subset. The final split contained 48, 17, and 17 patients, respectively, and the same patient was consistently assigned to a single subset across all magnification levels.

No explicit class balancing procedures were applied during dataset preparation. The original class distributions of both datasets were preserved during data partitioning. However, class imbalance during model training was addressed through a WeightedRandomSampler applied only to the training subset.

Table 2 summarizes the datasets used in this study, while Table 3 reports the corresponding BreaKHis partition statistics across magnification levels. The independent test cohort preserved the naturally imbalanced class distribution of the original dataset, with highly consistent class proportions across all magnification levels.

### 3.4. Model Architectures

In this study, three modern deep learning architectures were employed: ConvNeXt-Tiny, Swin-Tiny, and MaxViT-Tiny [4,5,6]. The Tiny variants were selected to ensure a fair comparison across architectures with comparable computational complexity while reflecting realistic deployment scenarios in computational pathology. In addition, all three architectures provide publicly available ImageNet-pretrained weights, enabling a consistent transfer-learning framework.

The selected models represent three complementary design paradigms. ConvNeXt-Tiny [4] is a modernized convolutional neural network that preserves the strengths of convolutional feature extraction while incorporating several design principles inspired by transformer architectures. Swin-Tiny [5] is a hierarchical vision transformer based on shifted-window self-attention, allowing efficient modeling of contextual relationships across image regions. MaxViT-Tiny [6] combines convolutional operations with transformer-based attention mechanisms, integrating local feature extraction and global context modeling within a unified architecture.

Together, these architectures provide a representative comparison of convolutional, transformer-based, and hybrid CNN–Transformer approaches, enabling investigation of how different feature representation mechanisms influence adaptation outcomes and false-negative rates under domain-shift conditions.

### 3.5. Adaptation Strategies

To systematically investigate the influence of adaptation depth on model behavior under realistic cross-domain conditions, three transfer-learning strategies were evaluated: zero-shot transfer, head-only fine-tuning, and full fine-tuning. These strategies represent increasing levels of adaptation complexity and provide a framework for analyzing the trade-off between computational requirements, adaptation flexibility, and sensitivity-oriented performance.

Zero-shot transfer:

In the zero-shot setting, models trained on the source-domain dataset were directly applied to the target BreaKHis dataset without additional optimization. No model parameters were updated during transfer. This strategy represents a challenging deployment scenario in which pretrained models are required to generalize to previously unseen data distributions and acquisition conditions without exposure to target-domain information.

Head-only fine-tuning:

For head-only fine-tuning, pretrained backbone parameters remained frozen while only the final classification layers were updated using target-domain training data. This lightweight adaptation strategy preserves previously learned feature representations while requiring only limited computational resources. Such an approach may be particularly relevant for deployment-oriented scenarios where rapid adaptation is required and extensive retraining may not be feasible.

Full fine-tuning:

In the full fine-tuning setting, all network parameters were optimized using target-domain training data. Unlike head-only adaptation, this strategy enables modification of both low-level and high-level feature representations, potentially improving robustness when substantial domain differences exist between source and target datasets.

All strategies were evaluated under identical patient-level separation conditions and using the same experimental protocol to ensure fair comparison across architectures and adaptation settings. Comparative analysis of these strategies enables investigation of how adaptation depth influences robustness, magnification-specific behavior, and diagnostic error profiles.

**Figure 2:**
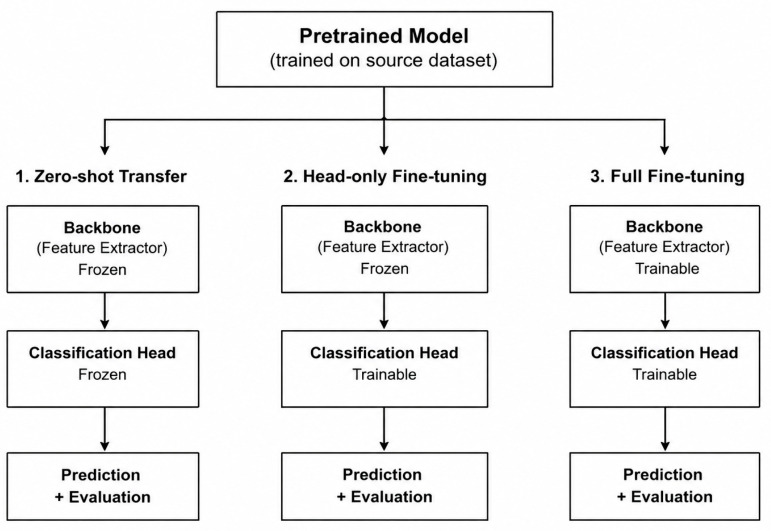
Overview of the adaptation strategies evaluated in this study. Starting from a pretrained source-domain model, three adaptation settings were investigated: zero-shot transfer without parameter updates, head-only fine-tuning where only the classification layers were updated, and full fine-tuning where all model parameters were optimized using target-domain data.

A visual summary of the evaluated adaptation strategies is presented in Figure 2, illustrating the differences in parameter updating and adaptation scope across the investigated settings.

### 3.6. Implementation Details

All experiments were implemented using the PyTorch 2.2 deep learning framework. ConvNeXt-Tiny, Swin-Tiny, and MaxViT-Tiny were initialized with ImageNet-pretrained weights and subsequently adapted to the target domain according to the transfer strategies described in Section 3.5.

Input images were resized to 224 × 224 pixels and normalized using ImageNet statistics (mean = [0.485, 0.456, 0.406], standard deviation = [0.229, 0.224, 0.225]). Data augmentation was applied exclusively to the training subset and included random horizontal and vertical flips together with random rotations within ±15°. Validation and test images remained unchanged.

Model optimization employed AdamW (weight decay = 0.01) with a batch size of 32. A fixed random seed of 42 was used for Python 3.9.0, NumPy 1.26.4, and PyTorch 2.2 operations to improve reproducibility. No learning-rate scheduler was employed. Identical preprocessing, augmentation, and optimization settings were used across all experiments to ensure fair comparison.

A learning rate of 1 × 10^−4^ was employed for both head-only adaptation and full fine-tuning across all evaluated architectures. To address class imbalance, a WeightedRandomSampler was applied exclusively to the training subset while preserving the original class distributions of the validation and test sets.

For source-domain training on the Kaggle dataset, all architectures were trained for three epochs. This duration was selected because validation performance saturated rapidly and additional training provided limited improvement relative to the associated computational cost. Model selection was based on validation ROC–AUC, and the final source-domain models corresponded to the checkpoints achieving the highest validation ROC–AUC prior to transfer.

For adaptation experiments, training was performed for a maximum of eight epochs with early stopping (patience = 2) based on validation ROC–AUC. The reported results correspond to the checkpoint achieving the highest validation ROC–AUC rather than the final training epoch.

For adapted models (head-only and full fine-tuning), two decision-threshold settings were evaluated. The conventional setting used a probability threshold of 0.5, whereas a validation-based threshold was additionally optimized to prioritize malignant case detection while maintaining acceptable specificity.

Threshold optimization was performed using a grid search over values ranging from 0.05 to 0.95 with a step size of 0.01. For each architecture and adaptation strategy, a single threshold was optimized using the complete patient-level validation set containing images from all magnification levels. Consequently, separate thresholds were not derived for individual magnification levels. The resulting validation-derived threshold was subsequently applied unchanged to all magnification-specific test evaluations.

For each candidate threshold, sensitivity, specificity, F1-score, and false-negative rate were computed on the validation set. The selected threshold maximized sensitivity subject to a minimum specificity constraint of 70%. When multiple thresholds achieved identical sensitivity values, preference was given to the threshold with higher specificity and subsequently higher F1-score. If no threshold satisfied the specificity constraint, the threshold achieving the highest F1-score was selected. The resulting threshold was then applied unchanged to the independent test set. Optimized threshold values are reported and discussed in Section 5.4, whereas zero-shot experiments retained the conventional threshold of 0.5.

This procedure was designed to reduce missed malignant cases while avoiding an unconstrained increase in false-positive predictions. For this reason, the optimized thresholds should be interpreted as validation-derived operating points rather than universally applicable diagnostic thresholds.

Because threshold optimization was performed on a patient-level validation cohort containing 17 patients, the selected thresholds may be influenced by patient-specific characteristics present within the validation set. Accordingly, these thresholds should be regarded as dataset-specific operating points and interpreted with appropriate caution.

All experiments were conducted on a CPU-based workstation equipped with an Intel Core i7-8700 processor (Intel Corporation, Santa Clara, CA, USA) operating at 3.20 GHz and 32 GB RAM. Although CPU training required longer execution times, this configuration enabled fully reproducible evaluation under resource-constrained conditions.

The adopted training protocol was designed to balance adaptation capability, computational efficiency, and diagnostic performance while ensuring consistent comparison across architectures, adaptation strategies, and magnification levels.

### 3.7. Evaluation Metrics

To quantitatively assess model performance in the cross-domain setting, several widely used classification metrics were employed, including accuracy, precision, recall (sensitivity), F1-score, Receiver Operating Characteristic Area Under the Curve (ROC–AUC), and Precision–Recall Area Under the Curve (PR–AUC) [26]. Together, these metrics provide complementary insights into classification performance, diagnostic error profiles, and robustness under domain shift.

Let

TP = true positives

TN = true negatives

FP = false positives

FN = false negatives

(1) $$Accuracy=\frac{TP+TN}{TP+TN+FP+FN}$$

(2) $$Precision=\frac{TP}{TP+FP}$$

(3) $$Recall=\frac{TP}{TP+FN}$$

(4) $$F1=2\cdot \frac{Precision\times Recall}{Precision+Recall}$$

The False-Negative Rate (FNR), representing the proportion of malignant cases incorrectly classified as benign, was additionally considered because missed malignant samples may directly affect diagnostic reliability and clinical decision-making.

(5) $$FNR=\frac{FN}{TP+FN}=1-Recall$$

ROC–AUC measures the ability of a classifier to discriminate between classes across all decision thresholds and is defined as:

(6) $$ROC\text{–}AUC=\int_{0}^{1}TPR(FPR)\;dFPR$$

where

(7) TPR=Recall

(8) $$FPR=\frac{FP}{FP+TN}$$

In Equation (6), dFPR represents the infinitesimal change in the false-positive rate used as the integration variable for calculating the area under the ROC curve.

PR–AUC is defined as:

(9) $$PR\text{–}AUC=\int_{0}^{1}Precision(Recall)\;dRecall$$

In Equation (9), dRecall denotes the infinitesimal change in recall used as the integration variable for calculating the area under the precision–recall curve. Unlike ROC–AUC, which evaluates discrimination capability across all decision thresholds, PR–AUC focuses on the trade-off between precision and recall and is particularly informative in imbalanced classification problems because it emphasizes performance on the positive class.

The selected metrics provide complementary perspectives on model performance. ROC–AUC and PR–AUC characterize global discrimination capability, whereas sensitivity, specificity, and false-negative rates provide additional insight into diagnostic error profiles.

From a diagnostic perspective, reduced sensitivity increases the risk of missed malignant cases, whereas reduced specificity may lead to unnecessary follow-up examinations and diagnostic interventions. Consequently, sensitivity and specificity should be interpreted jointly according to the intended clinical objective.

### 3.8. Statistical Validation Procedure

To evaluate whether the observed differences reflected systematic behavior rather than random variation, statistical comparisons were performed using the paired Wilcoxon signed-rank test [27] together with Cliff’s delta effect size [28]. The Wilcoxon signed-rank test was selected because it does not assume normally distributed paired observations and is suitable for comparative analysis of model performance across multiple experimental conditions.

(10) $$W=\sum_{i=1}^{n}R_{i}$$

where $R_{i}$ denotes the signed rank associated with the i-th paired difference and n represents the number of paired observations [27].

Cliff’s delta was additionally employed to quantify the magnitude of observed differences independently of statistical significance:

(11) $$\delta =\frac{\#(x_{i}\;>\;x_{j})\;-\;\#(x_{i}\;<\;x_{j})}{mn}$$

where m and n denote the sample sizes of the compared groups [28].

While the Wilcoxon signed-rank test determines whether observed differences are statistically significant, Cliff’s delta provides complementary information regarding the practical magnitude of those differences.

Effect-size magnitude was interpreted according to standard thresholds: negligible (∣δ∣ < 0.147), small (0.147 ≤ ∣δ∣ < 0.33), moderate (0.33 ≤ ∣δ∣ < 0.474), and large (∣δ∣ ≥ 0.474) [28].

To account for multiple pairwise comparisons, Holm correction was additionally applied to the obtained *p*-values. Statistical significance was considered at p<0.05.

To further assess the uncertainty associated with the patient-level test cohort, patient-level bootstrap resampling was additionally performed. Bootstrap resampling was performed at the patient level, with patients sampled with replacement while preserving all images corresponding to each selected patient. A total of 1000 bootstrap iterations were performed. For each iteration, sensitivity, false-negative rate, and ROC–AUC were recalculated, and 95% confidence intervals were estimated using the 2.5th and 97.5th percentiles of the resulting bootstrap distributions.

## 4. Results

### 4.1. Source-Domain Performance

The source-domain evaluation was conducted on the Kaggle breast histopathology dataset used during the initial model development stage. Three architectures, namely ConvNeXt-Tiny, Swin-Tiny, and MaxViT-Tiny, were selected for subsequent analysis because of their complementary architectural characteristics and their ability to represent different feature extraction mechanisms. Table 4 summarizes the classification performance obtained on the source-domain test set.

All evaluated architectures demonstrated strong in-domain classification performance across all evaluated metrics. ROC–AUC values remained consistently high, ranging from 0.965 to 0.967, indicating effective discrimination between benign and malignant tissue patterns. Similarly, all models achieved sensitivity values above 91%, suggesting reliable identification of malignant cases under source-domain conditions.

Among the evaluated architectures, Swin-Tiny achieved the highest sensitivity (94.17%), potentially indicating enhanced detection of malignant samples, whereas ConvNeXt-Tiny achieved the highest specificity (89.85%), suggesting a more balanced discrimination capability between benign and malignant categories. MaxViT-Tiny demonstrated intermediate performance characteristics, with results close to those of the other evaluated models. Swin-Tiny also achieved the highest PR–AUC (0.913), further supporting its strong malignant detection capability under source-domain conditions. Despite these differences, all three architectures achieved comparable overall performance under source-domain conditions.

The relatively similar source-domain performance across architectures suggests that all selected models successfully learned discriminative histopathological representations. However, in-domain evaluation alone cannot determine whether these representations remain effective after transfer to independent datasets. Therefore, additional experiments on BreaKHis were performed to assess transferability under dataset shift.

### 4.2. Zero-Shot Transfer Performance

To evaluate cross-domain generalization capability, the source-domain models were directly applied to the BreaKHis dataset without performing additional adaptation or fine-tuning. This experimental setting represents a zero-shot transfer scenario and enables assessment of model robustness under distributional differences between the source and target domains.

As shown in Table 5, all evaluated architectures experienced marked performance degradation after transfer to the target dataset. Compared with the strong source-domain results presented in Section 4.1, considerable reductions were observed across all evaluated metrics, particularly for malignant detection performance.

From a diagnostic perspective, the most important observation concerns the substantial increase in missed malignant cases. ConvNeXt-Tiny exhibited false-negative rates ranging from 59.95% to 91.07%, while Swin-Tiny and MaxViT-Tiny frequently exceeded 80% across multiple magnification levels. Such behavior indicates that a large proportion of malignant samples would be incorrectly classified as benign under direct deployment conditions.

Interestingly, although MaxViT-Tiny achieved comparatively higher ROC–AUC values in several zero-shot scenarios, its sensitivity remained extremely low. As a result, many malignant cases were still missed despite moderate discrimination performance. These findings further highlight the limited transferability of source-domain representations and reinforce the need for target-domain adaptation.

### 4.3. Head-Only Adaptation Results

Following the substantial performance degradation observed under direct zero-shot deployment, head-only adaptation was investigated as a lightweight transfer strategy aimed at improving cross-domain generalization while preserving previously learned feature representations. In this setting, only the classification layers were updated while pretrained backbone parameters remained frozen.

As shown in Table 6, head-only adaptation substantially improved performance across all evaluated architectures and magnification levels. Improvements were particularly evident in clinically relevant error measures, indicating enhanced malignant detection capability after adaptation.

ConvNeXt-Tiny showed relatively stable performance across magnification levels, with sensitivity values ranging from 89.29% to 93.70% and FN rates between 6.30% and 10.71%. MaxViT-Tiny achieved similar results, maintaining sensitivity above 92% across all magnification levels while keeping FN rates below 8%.

Among all evaluated configurations, Swin-Tiny achieved the strongest malignant detection performance within the investigated setting. Sensitivity remained above 96% across all magnification levels and reached 98.24% at 100× magnification, while FN rates remained consistently low, ranging from 1.76% to 3.87%. These results were obtained despite ROC–AUC values that were not consistently the highest among the evaluated models, indicating that strong malignant detection performance does not necessarily coincide with the highest discrimination scores.

Head-only adaptation considerably improved performance relative to zero-shot transfer while requiring only limited parameter updates, suggesting an attractive trade-off between computational efficiency and diagnostic performance.

### 4.4. Full Fine-Tuning Results

Following the head-only experiments, full fine-tuning was investigated to assess whether updating all network parameters could further improve cross-domain adaptation performance. In this setting, both the feature extraction backbone and classification layers were optimized using target-domain training data.

As shown in Table 7, full fine-tuning maintained strong results across all evaluated architectures and magnification levels. Sensitivity remained above 91% for all configurations, while false-negative rates stayed below 9%, representing a substantial improvement relative to direct zero-shot transfer.

ConvNeXt-Tiny remained relatively stable across magnification levels, achieving sensitivity values between 91.37% and 95.47%. Swin-Tiny produced comparable results, with peak sensitivity reaching 96.22% at 100× magnification. MaxViT-Tiny achieved the highest ROC–AUC values among the evaluated configurations, reaching 0.888 at 40× magnification and remaining above 0.80 across all magnification levels.

Despite its strong overall performance, full fine-tuning did not consistently outperform head-only adaptation across all evaluation measures. In several cases, head-only adaptation achieved comparable or more favorable malignant detection results, indicating that increased adaptation complexity does not always translate into improved clinical performance.

Full fine-tuning substantially improved cross-domain robustness compared with direct zero-shot transfer and provided strong overall discrimination capability. However, the results also indicate that increasing adaptation complexity does not always produce proportional improvements in malignant case detection performance.

### 4.5. Comparative Analysis of Adaptation Strategies

To facilitate comparison across architectures and adaptation strategies, mean performance values were computed across the four BreaKHis magnification levels (40×, 100×, 200×, and 400×). This aggregation provides a global view of adaptation behavior while reducing the influence of magnification-specific variability. Table 8 summarizes the resulting performance trends.

Both adaptation strategies substantially improved results relative to zero-shot transfer. Across all architectures, direct transfer produced extremely high false-negative rates (75.85–90.11%), whereas head-only adaptation and full fine-tuning consistently reduced the proportion of missed malignant cases to below 10%. These findings highlight the importance of adaptation when transferring models between independent histopathology datasets.

Differences between the two approaches were generally modest. Full fine-tuning often produced slightly higher specificity and more balanced overall behavior, whereas head-only adaptation frequently achieved comparable sensitivity while yielding fewer missed malignant cases. The most favorable configuration was Swin-Tiny with head-only adaptation, which achieved a mean sensitivity of 97.36% and a mean false-negative rate of 2.64%, compared with 5.32% for the corresponding full fine-tuning model.

A different trend was observed for MaxViT-Tiny, which achieved the highest mean ROC–AUC value (0.849) after full fine-tuning but did not produce the lowest false-negative rate. These results indicate that discrimination performance and malignant case detection do not always improve simultaneously after adaptation.

The results demonstrate that both architecture design and adaptation depth influence performance after transfer to the target domain.

**Figure 3:**
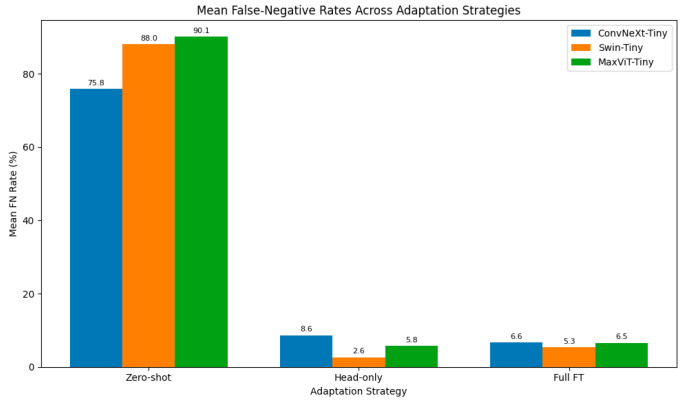
Mean false-negative rates across adaptation strategies for ConvNeXt-Tiny, Swin-Tiny, and MaxViT-Tiny.

To further illustrate the effect of adaptation depth, Figure 3 summarizes the mean false-negative rates across all evaluated architectures and transfer strategies. The results demonstrate a substantial reduction in missed malignant cases after adaptation, highlighting the importance of target-domain adaptation when transferring models between independent datasets.

**Figure 4:**
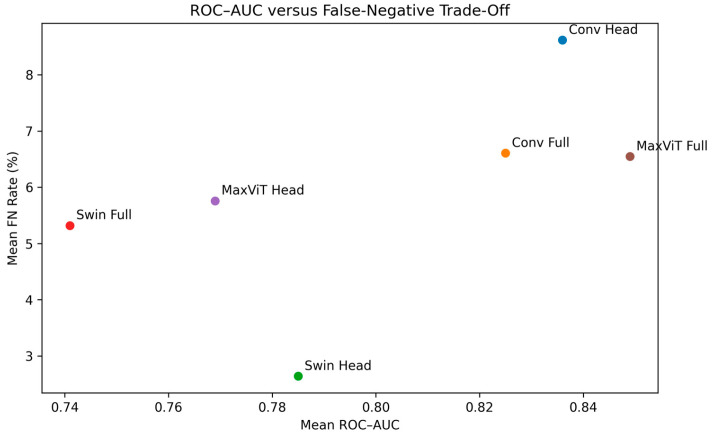
Relationship between mean ROC–AUC and false-negative rates for the adapted models. Zero-shot results were excluded from the analysis.

To further investigate the relationship between global discrimination capability and diagnostic error profiles, Figure 4 illustrates the trade-off between mean ROC–AUC and false-negative rates across the evaluated adaptation configurations. The results indicate that improvements in discrimination performance were not consistently accompanied by reductions in missed malignant cases.

**Figure 5:**
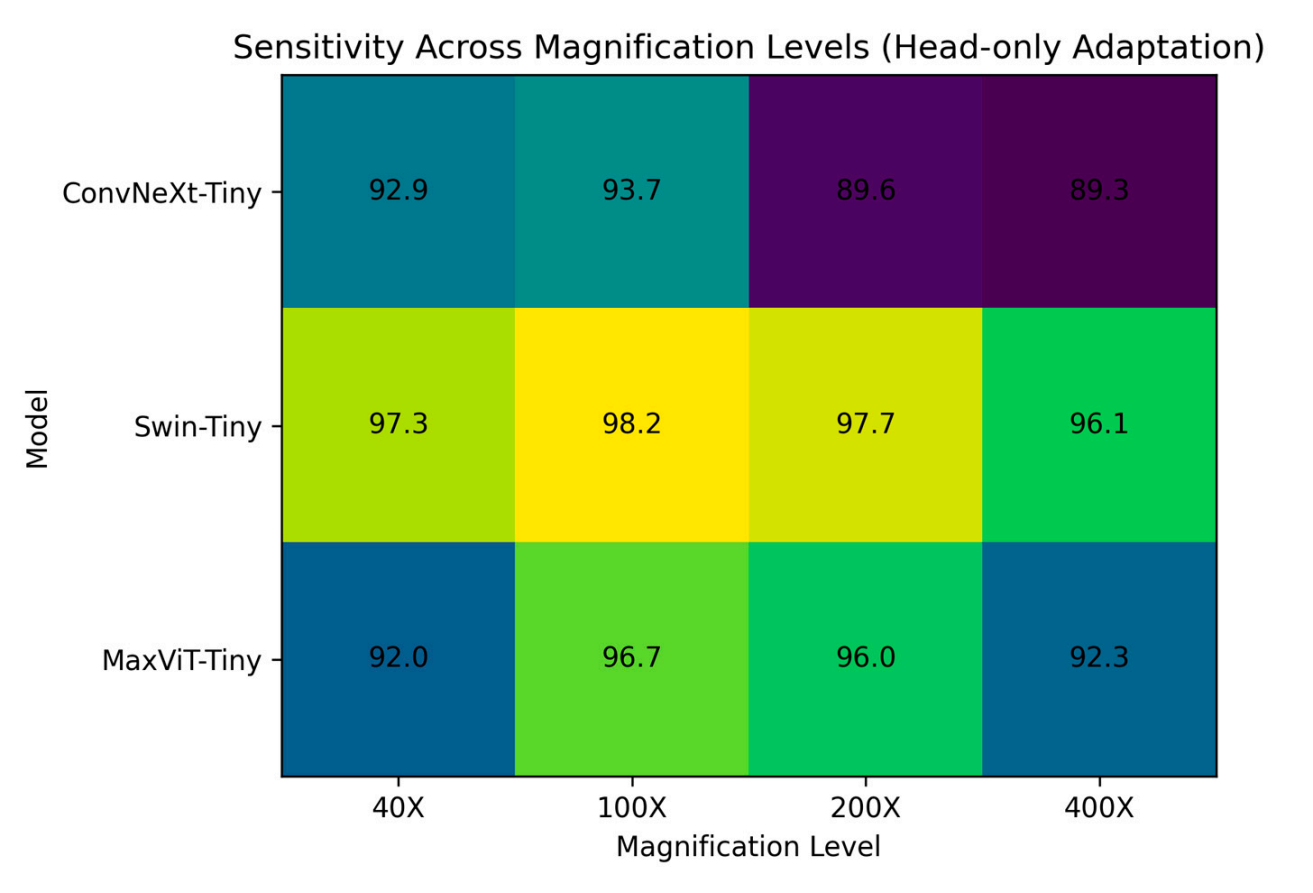
Sensitivity across BreaKHis magnification levels for head-only adaptation.

To further investigate model behavior across different image scales, Figure 5 summarizes sensitivity values across BreaKHis magnification levels for the head-only adaptation setting using a heatmap representation. Swin-Tiny demonstrated consistently high sensitivity across all magnification levels, whereas ConvNeXt-Tiny and MaxViT-Tiny exhibited greater variability, suggesting architecture-dependent robustness under domain-shift conditions.

### 4.6. Sensitivity-Oriented Threshold Analysis

Beyond architectural adaptation, threshold optimization was investigated as an additional strategy for reducing false-negative errors. Standard evaluation employed the conventional decision threshold of 0.5, whereas adapted models were additionally evaluated using validation-optimized thresholds selected to prioritize sensitivity while maintaining acceptable specificity. The optimized thresholds varied considerably across architectures and adaptation strategies, indicating that the conventional threshold of 0.5 was not always appropriate under cross-domain conditions.

To quantify the effect of threshold optimization, the relative reduction in false-negative rates was calculated as:

(12) $$FN_{reduction}(\%)=\frac{FN_{0.5}-FN_{opt}}{FN_{0.5}}\times 100$$

where

- $FN_{0.5}$ denotes the mean false-negative rate obtained using the conventional threshold of 0.5;
- $FN_{opt}$ denotes the mean false-negative rate obtained using validation-optimized thresholds.

Table 9 and Table 10 compare conventional thresholding (0.5) with sensitivity-oriented operating thresholds to quantify the effect of decision-level adaptation on diagnostic error profiles.

Values were calculated from head-only adaptation experiments using conventional thresholding (0.5) and sensitivity-oriented operating thresholds across all magnification levels.

Threshold optimization led to lower false-negative rates and higher sensitivity across all evaluated architectures. The largest reduction was observed for Swin-Tiny, where the mean false-negative rate decreased from 19.38% to 2.64%, corresponding to an 86.38% relative reduction. ConvNeXt-Tiny and MaxViT-Tiny also demonstrated substantial reductions of 73.49% and 76.61%, respectively.

These improvements were achieved without modifying the underlying architectures or performing additional training, indicating that decision-level calibration can have meaningful impact on diagnostic error profiles. Although lower thresholds increased false-positive rates and reduced specificity, the resulting trade-off substantially improved malignant case detection across all evaluated models, suggesting that threshold optimization may represent a simple and computationally efficient complement to model adaptation when minimizing missed malignant cases is a primary objective.

### 4.7. Clinical Error Profile Analysis

Although ROC–AUC and F1-score provide useful information regarding discrimination capability, they do not fully characterize diagnostic error profiles. Therefore, an additional analysis focusing on sensitivity and false-negative rates was performed.

As summarized in Table 11, the evaluated architectures exhibited distinct clinical error profiles. The table highlights the best-performing configuration for each architecture and provides a concise summary of sensitivity, false-negative rates, and discrimination performance across the evaluated adaptation strategies.

These findings suggest that model selection may depend on the intended clinical objective. Configurations optimized for minimizing missed malignant cases may differ from those achieving the highest global discrimination performance.

To further improve transparency of the reported error profiles, Table 12 summarizes the aggregate TP, TN, FP, and FN counts obtained for the main adaptation configurations across the complete patient-level BreaKHis test cohort.

### 4.8. Statistical Analysis

To assess whether the observed differences between adaptation strategies reflected systematic behavior rather than random variation, paired Wilcoxon signed-rank tests were performed using magnification-specific results from the three evaluated architectures across four magnification levels (*n* = 12 paired observations per metric). Statistical comparisons were restricted to head-only adaptation and full fine-tuning because both represent target-domain adaptation strategies evaluated under comparable conditions. Zero-shot transfer was excluded because it consistently exhibited substantially degraded performance and does not involve target-domain adaptation.

Because the analysis was based on a limited number of paired observations, *p*-values should be interpreted with appropriate caution. Therefore, statistical testing was complemented by effect-size analysis and bootstrap-derived confidence intervals. Table 13 summarizes the comparison between head-only adaptation and full fine-tuning.

Statistical analysis revealed no significant differences between head-only adaptation and full fine-tuning for false-negative rate (*p* = 0.370), sensitivity (*p* = 0.380), or ROC–AUC (*p* = 0.677). In contrast, full fine-tuning achieved a statistically significant improvement in F1-score (*p* = 0.0049), although the corresponding effect size remained small-to-moderate (Cliff’s δ = −0.333).

To further assess result stability, patient-level bootstrap analysis was performed by resampling patients with replacement while preserving all images belonging to each selected patient. Table 14 summarizes bootstrap-derived 95% confidence intervals for sensitivity, false-negative rate, and ROC–AUC across the evaluated architectures and adaptation strategies.

The confidence intervals were generally consistent with the observed performance trends. Swin-Tiny under head-only adaptation maintained the highest sensitivity, achieving 97.41% (95% CI: 93.00–99.55%) together with the lowest false-negative rate of 2.59% (95% CI: 0.45–7.00%). In contrast, MaxViT-Tiny under full fine-tuning retained the highest ROC–AUC value of 0.846 (95% CI: 0.689–0.987). The confidence-interval analysis supported the stability of the reported findings despite the limited size of the patient-level test cohort.

### 4.9. Confidence and Uncertainty Analysis

To further investigate prediction reliability under domain-shift conditions, confidence and entropy characteristics were analyzed for the head-only adaptation setting. Correct and incorrect predictions were compared to determine whether uncertainty estimates could provide complementary information regarding model behavior and prediction reliability. Table 15 summarizes confidence and entropy characteristics for correct and incorrect predictions obtained under head-only adaptation.

As shown in Table 15, incorrect predictions generally exhibited higher entropy values than correctly classified samples across all evaluated architectures, indicating increased predictive uncertainty during misclassification events. Confidence values also tended to be lower for incorrectly classified samples, although the magnitude of separation varied across architectures.

ConvNeXt-Tiny demonstrated the clearest distinction between correct and incorrect predictions, with confidence decreasing from 0.759 to 0.729 and entropy increasing from 0.496 to 0.546. Swin-Tiny exhibited a moderate separation pattern, whereas MaxViT-Tiny showed only minimal differences, particularly in confidence values.

These results suggest that confidence and entropy measures capture certain differences between correct and incorrect predictions. However, the observed separations remained relatively small and were not consistent across architectures.

The present analysis should be interpreted as an exploratory assessment of prediction uncertainty. More comprehensive calibration analyses, including reliability diagrams, expected calibration error, Brier score, and coverage–risk evaluation, remain important directions for future work.

## 5. Discussion

### 5.1. Key Findings

The results highlight the challenges associated with transferring breast histopathology models between independent datasets. Although all evaluated architectures achieved strong source-domain performance, direct transfer to BreaKHis without adaptation resulted in severe performance degradation, particularly in terms of sensitivity and false-negative rate. These findings indicate that representations learned from the source dataset did not generalize reliably to the target data distribution.

The poor zero-shot performance is likely explained by both domain shift and task mismatch. The Kaggle source dataset consists of small IDC image patches, whereas BreaKHis contains benign and malignant breast tumor images acquired under multiple magnification levels and different imaging conditions. As a result, the transfer scenario combines differences in data distribution, image characteristics, and classification objectives, creating a particularly challenging generalization setting.

Both adaptation strategies substantially improved performance relative to zero-shot transfer. In addition, validation-based threshold optimization further increased sensitivity while maintaining acceptable specificity, indicating that decision-level calibration can complement model adaptation when the primary objective is to reduce missed malignant cases.

The evaluated architectures exhibited distinct performance characteristics. Swin-Tiny achieved the highest sensitivity and the lowest false-negative rates under head-only adaptation, whereas MaxViT-Tiny generally produced the strongest ROC–AUC values. ConvNeXt-Tiny provided a more balanced trade-off between sensitivity and specificity. These findings suggest that model selection should be guided by the intended objective rather than discrimination performance alone.

A particularly interesting observation emerged for Swin-Tiny. Although both head-only adaptation and full fine-tuning improved performance compared with zero-shot transfer, head-only adaptation achieved lower false-negative rates and higher sensitivity. The best full fine-tuning checkpoint was selected after the first training epoch according to validation ROC–AUC, suggesting that the pretrained Swin representations already captured features relevant to the target dataset and that additional parameter updates provided limited benefit. Nevertheless, larger target cohorts, alternative optimization schedules, and extended adaptation procedures may influence this relationship and warrant further investigation.

Statistical testing, effect-size analysis, and bootstrap confidence intervals provided additional evidence regarding the stability of the observed trends. However, statistically significant differences were observed only for F1-score. Consequently, the remaining findings should be interpreted as consistent performance patterns rather than definitive evidence of superiority between adaptation strategies.

### 5.2. Interpretation of Magnification-Dependent Behavior

Performance differences across magnification levels provide additional insight into how the evaluated architectures respond to changes in image scale. Although both adaptation strategies improved performance relative to zero-shot transfer, measurable variability remained across magnifications.

As illustrated in Figure 5, the architectures exhibited distinct sensitivity patterns during head-only adaptation. Swin-Tiny maintained consistently high sensitivity, remaining above 96% across all magnification levels, whereas ConvNeXt-Tiny showed greater variability, particularly at higher magnifications. MaxViT-Tiny achieved intermediate performance, with stronger results at 100× and 200× magnifications and moderate reductions at 40× and 400×.

These findings suggest that image scale influences feature extraction differently across architectures. Changes in magnification alter the balance between local cellular information and broader tissue context, which may affect representation quality and classification performance. The relatively stable behavior observed for Swin-Tiny may indicate that hierarchical attention mechanisms support more consistent feature learning across magnification levels, whereas the larger fluctuations observed for the other architectures suggest greater sensitivity to scale-dependent image characteristics. However, additional validation using larger and more diverse cohorts is required to confirm this interpretation.

Overall, the results indicate that magnification remains an important factor in breast histopathology classification and should be considered when evaluating model performance across heterogeneous imaging settings.

### 5.3. Comparison with Prior Work

To place the obtained findings in a broader context, Table 16 summarizes representative recent studies in breast histopathology classification and highlights methodological differences relative to the present work.

Recent studies have reported strong performance using convolutional neural networks, vision transformers, hybrid CNN–Transformer models, and magnification-aware learning strategies [13,29,30,31,32,33,34]. These approaches have demonstrated the effectiveness of modern deep learning methods for breast histopathology analysis and have contributed to substantial progress in the field.

Nevertheless, direct numerical comparisons should be interpreted with caution because datasets, partitioning strategies, and evaluation protocols differ considerably across studies. Most previous investigations focused on within-dataset evaluation, whereas the present work examines adaptation between independent datasets using patient-disjoint evaluation.

The comparison presented in Table 16 is therefore intended to provide methodological context rather than a direct ranking of competing approaches. In contrast to studies that primarily emphasize accuracy or ROC–AUC, the present work investigates how adaptation depth influences sensitivity and false-negative behavior after transfer to an external target dataset.

The results further suggest that different evaluation objectives may favor different architectures. While some configurations emphasized discrimination capability, others provided more favorable diagnostic error profiles after adaptation.

Overall, the reported results should be interpreted within the context of a more demanding evaluation setting involving independent datasets and patient-disjoint assessment. Moderate reductions in conventional performance metrics may therefore reflect increased evaluation rigor rather than inferior model quality.

### 5.4. Clinical Applicability and Deployment Scenarios

From a clinical perspective, reliable detection of malignant cases is a critical requirement for computational pathology systems because diagnostic misses may adversely affect patient management and treatment decisions. Consequently, sensitivity and clinically relevant error measures may provide valuable information beyond global discrimination metrics when evaluating breast histopathology models.

The results suggest that different deployment objectives may favor different adaptation strategies. Head-only adaptation frequently achieved high sensitivity while minimizing missed malignant cases, making it particularly attractive in settings where cancer detection is prioritized. In contrast, full fine-tuning generally produced a more balanced trade-off between sensitivity and specificity, although at the cost of increased computational requirements and broader parameter updates.

To further assess practical feasibility, Table 17 summarizes adaptation efficiency characteristics across architectures and adaptation strategies, including convergence behavior, computational cost, validation ROC–AUC, and optimized decision thresholds. Considerable differences were observed across configurations. ConvNeXt-Tiny head-only adaptation demonstrated the lowest computational cost, requiring approximately 40 min of CPU-based training while maintaining competitive validation performance. In contrast, MaxViT-Tiny full fine-tuning achieved the highest validation ROC–AUC (0.9953) but required the greatest computational cost (approximately 349 min). Swin-Tiny full fine-tuning converged after a single epoch while maintaining validation performance comparable to the corresponding head-only configuration. These observations suggest that improved discrimination performance may not always justify increased computational demands and that lightweight adaptation strategies may provide favorable cost–performance trade-offs.

The optimized thresholds differed considerably from the conventional value of 0.5, highlighting the importance of validation-based decision calibration. Lower thresholds generally reduced false-negative rates and improved malignant case detection, although often at the expense of specificity. Because threshold optimization was performed using a validation cohort containing 17 patients, the resulting operating points should be regarded as dataset-specific and interpreted with appropriate caution. Additional validation using larger cohorts and independent datasets would help assess threshold stability and generalizability.

Finally, confidence and uncertainty analysis revealed modest architecture-dependent differences in prediction reliability. Although the observed effects were limited, these findings suggest that uncertainty measures may provide complementary information and could support future uncertainty-aware decision-support strategies.

### 5.5. Limitations and Future Work

Several limitations should be acknowledged in the present study.

First, experiments were conducted using a single target dataset. Additional validation across multiple institutions and acquisition settings is therefore required to further assess generalizability. Accordingly, the reported findings should be interpreted as experimental evidence regarding adaptation performance rather than as direct clinical validation of an AI-assisted diagnostic system.

Second, the present study focused on patch-level breast histopathology classification rather than whole-slide image analysis. While patch-based evaluation provides a controlled setting for systematically comparing adaptation strategies, whole-slide workflows more closely reflect routine clinical practice and introduce additional challenges related to large-scale contextual information and computational complexity.

Third, all input images were resized to a fixed spatial resolution of 224 × 224 pixels to ensure compatibility with pretrained architectures. Although this preprocessing step enabled consistent comparisons across models, resizing may reduce fine-grained histopathological information and influence feature representation across magnification levels.

Fourth, although sensitivity-oriented measures were explicitly incorporated into the evaluation protocol, the present investigation primarily focused on predictive performance and adaptation outcomes. Future studies could include broader interpretability analyses, calibration assessment, uncertainty quantification, and uncertainty-aware decision strategies to further improve model reliability and confidence estimation.

An additional limitation is the mismatch between the source and target classification tasks. While both datasets address breast cancer malignancy assessment, the differences in labeling strategy and classification objectives may reduce transferability and contribute to the performance degradation observed during zero-shot transfer.

Another limitation concerns the relatively small patient-level evaluation cohort. Although strict patient-level separation was employed, the independent test subset contained only 17 patients. Consequently, the reported performance estimates may be influenced by the specific patient composition of the selected split. Future investigations should therefore consider repeated patient-level splits, cross-validation protocols, and larger multi-center cohorts to further assess robustness and generalizability.

Future work should further evaluate the proposed framework on larger multi-center cohorts and more diverse clinical datasets. Additional studies may investigate whole-slide image analysis, foundation-model-based representations, advanced adaptation techniques, and uncertainty-aware decision-support strategies.

## 6. Conclusions

This study introduced a patient-level cross-domain evaluation framework for breast histopathology classification and systematically compared multiple adaptation strategies across three modern deep learning architectures: ConvNeXt-Tiny, Swin-Tiny, and MaxViT-Tiny.

Direct zero-shot transfer resulted in severe performance degradation across all evaluated architectures, confirming the difficulty of transferring breast histopathology models between independent datasets without adaptation. Both adaptation strategies substantially improved performance. However, full fine-tuning did not consistently produce the most favorable clinical error profile. Swin-Tiny under head-only adaptation achieved the highest sensitivity (97.36%) and lowest false-negative rate (2.64%), whereas MaxViT-Tiny achieved the highest mean ROC–AUC (0.849) after full fine-tuning.

These findings demonstrate that the best-performing model may depend on the evaluation objective. Architectures achieving the strongest discrimination performance did not necessarily produce the lowest false-negative burden. Additional analyses further highlighted practical considerations related to computational cost, threshold optimization, and prediction uncertainty. ConvNeXt-Tiny head-only adaptation demonstrated the most computationally efficient behavior, whereas MaxViT-Tiny full fine-tuning required substantially greater computational resources.

Overall, the results emphasize the importance of complementing conventional discrimination metrics with clinically relevant error analysis when evaluating breast histopathology models. Sensitivity and false-negative behavior provide valuable information beyond ROC–AUC and accuracy, particularly in applications where minimizing missed malignant cases represents a primary objective.

The main contributions of this study can be summarized as follows:

1. We establish a patient-level cross-domain evaluation framework for breast histopathology classification that minimizes information leakage and provides more realistic estimates of model generalization.
2. We conduct a systematic comparison of ConvNeXt-Tiny, Swin-Tiny, and MaxViT-Tiny under identical adaptation protocols across multiple magnification levels.
3. We evaluate and compare zero-shot transfer, head-only adaptation, and full fine-tuning while analyzing their influence on malignant case detection and diagnostic error characteristics.
4. We demonstrate that models achieving the highest discrimination performance do not necessarily achieve the lowest false-negative rates after transfer to an external dataset.
5. We investigate threshold optimization, uncertainty characteristics, and computational efficiency to provide a broader perspective on practical model reliability and deployment.

## Figures and Tables

**Table 1:** Representative studies on breast histopathology classification, robustness, and deployment-related challenges.

Ref	Study Focus	Dataset	Architecture/Method	Evaluation Strategy	Main Findings	Limitations
[8]	Breast histopathology benchmark	BreaKHis	Dataset introduction	Image-level evaluation	Introduced a widely adopted multi-magnification benchmark	No patient-level protocol
[13]	Breast cancer diagnosis	Whole-slide histopathology images	Deep learning classification framework	Standard train/test	Demonstrated promising diagnostic performance	Limited deployment analysis
[14]	AI applications in breast pathology	Multiple studies	Literature review	Literature review	Summarized diagnostic and clinical applications of AI	No experimental validation
[15]	Stain normalization and robustness	Histopathological datasets	Deep learning normalization methods	Cross-dataset evaluation	Reduced color variability and improved robustness	Additional preprocessing complexity
[16]	Multi-center generalization	Multi-center histopathology datasets	Generalizable AI framework	Cross-center evaluation	Improved robustness across heterogeneous datasets	Increased training complexity
[17]	Breast histology classification	Breast histology images	CNN-based framework	Standard train/test	Demonstrated effective CNN-based diagnosis	Limited generalization analysis
[18]	Magnification-independent classification	BreaKHis	Deep CNN	Multi-magnification evaluation	Improved robustness across image magnifications	Image-level evaluation
[19,20]	Modern computational pathology	Large-scale whole-slide datasets	Weakly supervised and foundation models	Large-scale training	Improved feature representation and generalization	High computational requirements
[21,22]	Domain generalization and robustness	Histopathology datasets	Generalization and augmentation methods	External/cross-domain evaluation	Improved robustness under domain shift	Residual performance degradation
Present study	Cross-domain breast histopathology classification	Kaggle source dataset + patient-level BreaKHis	ConvNeXt-Tiny, Swin-Tiny, MaxViT-Tiny	Patient-level evaluation + zero-shot + head-only + full fine-tuning	Systematic investigation of adaptation depth under domain shift using sensitivity and false-negative-oriented evaluation	—

**Table 2:** Summary of datasets employed in this study.

Dataset	Images	Patients	Classes	Magnification Levels	Role
Kaggle breast histopathology dataset	277,524	279	IDC-negative/IDC-positive	Patch-based	Source-domain training
BreaKHis	7909	82	Benign/Malignant	40×, 100×, 200×, 400×	Target-domain adaptation and evaluation
BreaKHis test subset	3861	17	929 benign/2932 malignant	40×, 100×, 200×, 400×	Patient-level evaluation

**Table 3:** Patient-level BreaKHis partition statistics across magnification levels.

Magnification	Split	Benign	Malignant	Total
40×	Train	680	1443	2123
	Validation	252	481	733
	Test	226	676	902
100×	Train	699	1365	2064
	Validation	244	519	763
	Test	240	794	1034
200×	Train	678	1226	1904
	Validation	209	580	789
	Test	240	790	1030
400×	Train	673	1146	1819
	Validation	192	490	682
	Test	223	672	895

**Table 4:** Source-domain performance of the evaluated architectures on the Kaggle breast histopathology dataset.

Model	Accuracy (%)	Precision (%)	Sensitivity (%)	Specificity (%)	F1-Score (%)	ROC–AUC	PR–AUC
ConvNeXt-Tiny	90.24	75.29	91.39	89.85	82.56	0.967	0.910
Swin-Tiny	88.42	70.21	94.17	86.48	80.44	0.967	0.913
MaxViT-Tiny	89.97	74.75	91.11	89.59	82.13	0.965	0.908

**Table 5:** Zero-shot transfer performance on the BreaKHis dataset across all magnification levels.

Model	Magnification	Accuracy (%)	Sensitivity (%)	Specificity (%)	F1-Score (%)	ROC–AUC	PR-AUC	FN Rate (%)
ConvNeXt	40×	28.16	22.04	46.46	31.50	0.258	0.664	77.96
ConvNeXt	100×	37.14	40.05	27.50	49.46	0.262	0.638	59.95
ConvNeXt	200×	26.02	25.57	27.50	34.65	0.201	0.607	74.43
ConvNeXt	400×	21.45	8.93	59.19	14.58	0.260	0.621	91.07
Swin	40×	25.50	19.53	43.36	28.21	0.258	0.639	80.47
Swin	100×	22.05	12.59	53.33	19.88	0.222	0.626	87.41
Swin	200×	19.22	8.35	55.00	13.69	0.234	0.624	91.65
Swin	400×	23.58	7.44	72.20	12.76	0.209	0.616	92.56
MaxViT	40×	33.70	13.31	94.69	23.14	0.507	0.774	86.69
MaxViT	100×	27.47	10.83	82.50	18.66	0.410	0.730	89.17
MaxViT	200×	27.38	8.86	88.33	15.77	0.442	0.746	91.14
MaxViT	400×	24.25	6.55	77.58	11.49	0.385	0.670	93.45

**Table 6:** Head-only adaptation performance on the BreaKHis dataset across all magnification levels.

Model	Magnification	Sensitivity (%)	Specificity (%)	F1-Score (%)	ROC–AUC	PR-AUC	FN Rate (%)
ConvNeXt	40×	92.90	56.19	89.52	0.823	0.928	7.10
ConvNeXt	100×	93.70	61.67	91.29	0.827	0.922	6.30
ConvNeXt	200×	89.62	55.83	88.28	0.827	0.916	10.38
ConvNeXt	400×	89.29	56.95	87.72	0.867	0.955	10.71
Swin	40×	97.34	42.04	89.83	0.814	0.917	2.66
Swin	100×	98.24	46.67	91.66	0.771	0.896	1.76
Swin	200×	97.72	52.50	92.12	0.778	0.901	2.28
Swin	400×	96.13	34.98	88.31	0.779	0.888	3.87
MaxViT	40×	92.01	46.02	87.61	0.791	0.909	7.99
MaxViT	100×	96.73	54.58	91.92	0.781	0.903	3.27
MaxViT	200×	95.95	50.00	90.89	0.762	0.884	4.05
MaxViT	400×	92.26	55.61	89.14	0.740	0.845	7.74

**Table 7:** Full fine-tuning performance on the BreaKHis dataset across all magnification levels.

Model	Magnification	Sensitivity (%)	Specificity (%)	F1-Score (%)	ROC–AUC	PR-AUC	FN Rate (%)
ConvNeXt	40×	92.31	59.29	89.66	0.855	0.939	7.69
ConvNeXt	100×	95.47	59.17	91.88	0.810	0.911	4.53
ConvNeXt	200×	94.43	63.33	91.87	0.801	0.921	5.57
ConvNeXt	400×	91.37	60.54	89.37	0.836	0.934	8.63
Swin	40×	94.97	58.41	90.93	0.781	0.898	5.03
Swin	100×	96.22	62.50	92.72	0.751	0.871	3.78
Swin	200×	94.68	60.83	91.67	0.739	0.869	5.32
Swin	400×	92.86	49.33	88.57	0.692	0.808	7.14
MaxViT	40×	91.42	61.06	89.44	0.888	0.958	8.58
MaxViT	100×	95.21	61.25	92.03	0.838	0.933	4.79
MaxViT	200×	93.42	61.67	91.11	0.811	0.923	6.58
MaxViT	400×	93.75	58.74	90.39	0.859	0.946	6.25

**Table 8:** Mean performance comparison of transfer strategies across all BreaKHis magnification levels.

Model	Strategy	Sensitivity (%)	Specificity (%)	F1-Score (%)	ROC–AUC	PR-AUC	FN Rate (%)
ConvNeXt	Zero-shot	24.15	40.16	32.55	0.245	0.633	75.85
ConvNeXt	Head-only	91.38	57.66	89.20	0.836	0.930	8.62
ConvNeXt	Full FT	93.39	60.58	90.70	0.825	0.926	6.61
Swin	Zero-shot	11.98	55.97	18.63	0.231	0.626	88.02
Swin	Head-only	**97.36**	44.04	90.48	0.785	0.901	**2.64**
Swin	Full FT	94.68	57.77	**90.97**	0.741	0.862	5.32
MaxViT	Zero-shot	9.89	**85.78**	17.26	0.436	0.730	90.11
MaxViT	Head-only	94.24	51.55	89.89	0.769	0.885	5.76
MaxViT	Full FT	93.45	60.68	90.74	**0.849**	**0.940**	6.55

**Table 9:** Effect of sensitivity-oriented threshold optimization during head-only adaptation.

Model	Threshold Strategy	Mean Sensitivity (%)	Mean Specificity (%)	Mean F1-Score (%)	Mean FN Rate (%)	Mean FP Rate (%)
ConvNeXt	Standard (0.5)	67.49	80.34	77.05	32.52	19.65
ConvNeXt	Sensitivity-oriented	91.38	57.66	89.20	8.62	42.34
Swin	Standard (0.5)	80.62	66.51	84.25	19.38	33.49
Swin	Sensitivity-oriented	97.36	44.04	90.48	2.64	55.96
MaxViT	Standard (0.5)	75.37	67.90	81.21	24.63	32.10
MaxViT	Sensitivity-oriented	94.24	51.55	89.89	5.76	48.45

**Table 10:** Relative reduction in false-negative rates after threshold optimization.

Model	Mean FN Rate @0.5 (%)	Mean FN Rate Optimized (%)	Relative FN Reduction (%)
ConvNeXt	32.52	8.62	73.49
Swin	19.38	2.64	**86.38**
MaxViT	24.63	5.76	76.61

**Table 11:** Clinical error profile summary of the evaluated architectures.

Model	Best Strategy	Sensitivity (%)	FN Rate (%)	ROC–AUC	Clinical Interpretation
ConvNeXt	Full FT	93.39	6.61	0.825	Balanced behavior
Swin	Head-only	**97.36**	**2.64**	0.785	Lowest missed malignant cases
MaxViT	Full FT	93.45	6.55	**0.849**	Strong discrimination performance

**Table 12:** Aggregate confusion-matrix counts for the best-performing adaptation configurations on the patient-level BreaKHis test cohort.

Model	Best Strategy	TP	TN	FP	FN
ConvNeXt-Tiny	Full FT	2742	563	366	190
Swin-Tiny	Head-only	2856	411	518	76
MaxViT-Tiny	Full FT	2742	564	365	190

**Table 13:** Statistical comparison between head-only adaptation and full fine-tuning using paired Wilcoxon signed-rank analysis across magnification-specific results.

Comparison	Metric	*p*-Value	Cliff’s δ	Effect-Size Interpretation
Head-only vs. Full Fine-Tuning	FN Rate	0.370	−0.153	Negligible
Head-only vs. Full Fine-Tuning	Sensitivity	0.380	0.153	Negligible
Head-only vs. Full Fine-Tuning	ROC–AUC	0.677	−0.167	Negligible
Head-only vs. Full Fine-Tuning	F1-score	**0.0049**	−0.333	Small–Moderate

**Table 14:** Patient-level bootstrap 95% confidence intervals for clinically relevant performance metrics.

Model	Strategy	Patients	Images	Sensitivity (95% CI)	FN Rate (95% CI)	ROC–AUC (95% CI)
ConvNeXt-Tiny	Head-only	17	3861	91.41 (80.95–99.39)	8.59 (0.61–19.05)	0.831 (0.646–0.964)
ConvNeXt-Tiny	Full FT	17	3861	93.52 (81.47–99.12)	6.48 (0.88–18.53)	0.823 (0.710–0.988)
Swin-Tiny	Head-only	17	3861	97.41 (93.00–99.55)	2.59 (0.45–7.00)	0.788 (0.599–0.956)
Swin-Tiny	Full FT	17	3861	94.75 (85.28–99.18)	5.25 (0.82–14.72)	0.746 (0.601–0.980)
MaxViT-Tiny	Head-only	17	3861	94.41 (87.17–98.58)	5.59 (1.42–12.83)	0.773 (0.619–0.956)
MaxViT-Tiny	Full FT	17	3861	93.52 (84.61–99.84)	6.48 (0.16–15.39)	0.846 (0.689–0.987)

**Table 15:** Confidence and Uncertainty Characteristics for Head-only Adaptation.

Model	Mean Confidence (Correct)	Mean Confidence (Incorrect)	Mean Entropy (Correct)	Mean Entropy (Incorrect)
ConvNeXt-Tiny	0.759	0.729	0.496	0.546
Swin-Tiny	0.787	0.777	0.457	0.492
MaxViT-Tiny	0.752	0.751	0.499	0.515

**Table 16:** Comparison with representative recent breast histopathology studies.

Study	Method	Dataset/Protocol	Reported Metrics	Focus
Rafiq et al. [13]	DenseNet121	Histopathology images	Accuracy = 98.50%; AUC = 0.98	Binary and multi-class classification
Abimouloud et al. [29]	Token Vision Transformer	Histopathology datasets	High classification performance	Efficient transformer design
Yan et al. [30]	DWNAT-Net	BreaKHis	Accuracy = 99.66%	Transformer + wavelet features
Liu and Min [31]	DCS-ST	Breast histopathology with limited annotations	Improved AUC/F1 performance	Cross-scale Swin Transformer
Baroni et al. [32]	Vision Transformer	Breast histopathology	Improved performance through pretraining and normalization	Cross-scale Swin Transformer
Kolla et al. [33]	Magnification-independent framework	BreaKHis	Strong classification performance across magnifications	Multi-scale robustness
Abimouloud et al. [34]	CNN + Vision Transformer	Breast histopathology	Improved classification performance	Hybrid representation learning
**This study**	ConvNeXt/Swin/MaxViT	Kaggle→BreaKHis, strict patient-level	Sensitivity = 97.36%; ROC–AUC = 0.849; FN Rate = 2.64%	FN-oriented clinical adaptation

**Table 17:** Adaptation Efficiency, Computational Cost, and Optimized Decision Thresholds Across Adaptation Strategies.

Model	Strategy	Best Epoch	Training Time (min)	Validation ROC–AUC	Optimized Threshold
ConvNeXt-Tiny	Head-only	2	40.0	0.9886	0.24
ConvNeXt-Tiny	Full FT	4	145.3	0.9916	0.05
Swin-Tiny	Head-only	8	105.7	0.9889	0.16
Swin-Tiny	Full FT	1	84.7	0.9879	0.05
MaxViT-Tiny	Head-only	8	184.9	0.9754	0.24
MaxViT-Tiny	Full FT	4	349.4	0.9953	0.06

## Data Availability

The original contributions presented in this study are included in the article, further inquiries can be directed to the corresponding author.

## Abbreviations

The following abbreviations are used in this manuscript:

AUC	Area Under the Curve
CAD	Computer-Aided Diagnosis
CNN	Convolutional Neural Network
FN	False Negative
FNR	False-Negative Rate
FP	False Positive
FT	Fine-Tuning
H&E	Hematoxylin and Eosin
IDC	Invasive Ductal Carcinoma
MIL	Multiple Instance Learning
PR–AUC	Precision–Recall Area Under the Curve
ROC	Receiver Operating Characteristic
ROC–AUC	Receiver Operating Characteristic Area Under the Curve
TP	True Positive
TN	True Negative
ViT	Vision Transformer
WSI	Whole-Slide Image
